# Therapeutic Efficacy of the Novel Stimuli-Sensitive Nano-Ferritins Containing Doxorubicin in a Head and Neck Cancer Model

**DOI:** 10.3390/ijms18071555

**Published:** 2017-07-18

**Authors:** Verena Damiani, Elisabetta Falvo, Giulio Fracasso, Luca Federici, Martina Pitea, Vincenzo De Laurenzi, Gianluca Sala, Pierpaolo Ceci

**Affiliations:** 1 Department of Medical, Oral and Biotechnological Sciences and CeSI-Met Centro Scienze dell’Invecchiamento e Medicina Traslazionale, Universita “G. d’Annunzio” di Chieti-Pescara, Chieti 66100, Italy; verena.damiani@unich.it (V.D.); lfederici@unich.it (L.F.); delaurenzi@unich.it (V.D.L.); 2 Institute of Molecular Biology and Pathology, National Research Council of Italy (CNR), Rome 00185, Italy; elisabetta.falvo@uniroma1.it (E.F.); pitea.1723539@studenti.uniroma1.it (M.P.); 3 Department of Medicine, University of Verona, Verona 37134, Italy; giulio.fracasso@univr.it; 4 Department of Biochemical Sciences “A. Rossi Fanelli”, Sapienza University of Rome, Rome 00185, Italy

**Keywords:** pasylated ferritin, stimuli-sensitive peptides, doxorubicin, drug-delivery, head and neck cancer

## Abstract

Doxorubicin is employed alone or in combination for the treatment of several hematological and solid malignancies; despite its efficacy, there are associated cardiotoxicity limits both in its application in patients with heart disease risk factors and also in its long-term use. HFt-MP-PAS40 is a genetically engineered human ferritin heavy chain (HFt)-based construct able to efficiently entrap and deliver doxorubicin to cancer cells. HF-MP-PAS contains a short motif sequence (defined as MP) responsive to proteolytic cleavage by tumor matrix metalloproteases (MMPs), located between each HFt subunit and a masking polypeptide sequence rich in proline (P), alanine (A), and serine (S) residues (PAS). This carrier displayed excellent therapeutic efficacy in a xenogenic pancreatic cancer model in vivo, leading to a significant increase in overall animal survival in treated mice. Herein, we describe the HFt-MP-PAS40-Dox efficacy against squamous cell carcinomas of the head and neck (HNSCC) with the goal of validating the application of our nano-drug for the treatment of different solid tumors. In addition, a tolerability study in healthy mice was also performed. The results indicate that HFt-MP-PAS40-Dox produced increased anti-tumor effects both in vitro and in vivo in comparison to the free drug in several HNSCC cell lines. In the acute toxicity studies, the maximum tolerated dose (MTD) of HFt-MP-PAS40-Dox was about 3.5 higher than the free drug: 25 mg/kg versus 7 mg/kg doxorubicin equivalents. Importantly, evaluation of heart tissues provided evidence that doxorubicin is less cardio-toxic when encapsulated inside the ferritin carrier. In conclusion, HFt-MP-PAS40-Dox may be administered safely at higher doses compared with the free drug, resulting in superior efficacy to control HNSCC malignancies.

## 1. Introduction

Human ferritin heavy chain (HFt)-based carriers have been shown to efficiently deliver chemotherapeutics to cancer cells [1,2,3,4,5,6,7,8]. HFt is a cage-shaped protein formed by 24 identical subunits with external and internal diameters of 12 and 8 nm, respectively [9,10,11,12,13,14,15]. An important advantage of the HFt system is the ability to be internalized by many types of tumor cells via the transferrin receptor 1 (TfR1). It was reported that a wide number of tumors express up to 100 times higher levels of TfR1 than healthy cells and actively internalize the HFt:TfR1 ligand-receptor complex to accumulate large amounts of iron required for the growth of cancer cells [16,17].

Recently, we have developed a novel HFt-based construct, named HFt-MP-PAS40, suitable for drug delivery. This was dictated by the necessity of increasing both the in vivo half-life of native HFt and the stability of HFt-drug complexes produced. This construct is characterized by an N-terminus of each HFt subunit genetically fused to: (i) a PAS polypeptide sequence of 40 residues rich in proline (P), alanine (A), and serine (S) residues [4,18]; and (ii) a short motif sequence (defined as MP) responsive to proteolytic cleavage by tumor matrix metalloproteases (MMPs), inserted between each HFt subunit and the outer PAS polypeptide. The PAS shield was used as an alternative to the less homogenous and more expensive polyethylene glycol (PEG)-based polymers. The presence of the MP sequence allows the PAS shield to be selectively removed by a specific stimulus present in the tumor microenvironment so that the resulting unmasked HFt can freely be internalized by TfR1 overexpressed in tumor cells.

The HFt-MP-PAS40 construct was able to encapsulate, in the internal cavity, three times more doxorubicin (Dox) than wild-type HFt, forming more stable complexes with higher in vivo circulation time. Significantly, Dox-loaded HFt-MP-PAS40 (HFt-MP-PAS40-Dox) displayed excellent antineoplastic properties in vitro towards human sarcoma and pancreatic adenocarcinoma cells, and also human pancreatic xenotransplants in vivo [18].

The goal of the present study was to expand our preclinical studies on the HFt-MP-PAS40-Dox, evaluating it on head and neck cancers. This type of cancer is often referred to as squamous cell carcinomas of the head and neck (HNSCC), because it begins in the squamous cells that line the mucosal surfaces inside the head and neck. Head and neck cancers account for approximately 4% of all cancers in the United States [19]. The median overall survival for patients with recurrent and/or metastatic (R/M) HNSCC remains < 1 year despite modern systemic chemotherapy and targeted agents. Cisplatin-based chemotherapy is an important part of the multimodality treatment for HNSCC [20,21]. However, the effectiveness of cisplatin-based monotherapy is far from being satisfactory. In the present study, we report the use of HFt-MP-PAS40-Dox as an anticancer therapy in a xenograft mouse model with the goal of improving the treatment of human HNSCC.

## 2. Results and Discussion

### 2.1. Dox Encapsulation in HFt-MP-PAS40

Dox encapsulation inside HFt-MP-PAS40 was achieved following the disassembly/reassembly procedure reported previously [4]. The encapsulation efficiency using this procedure allowed about 90 molecules of Dox to be entrapped inside the protein cage, that is a value approximately three times higher than the native HFt. This higher yield was mainly ascribed to the stabilizing effect of the PAS polymer toward the extreme conditions required by the pH-jump used to dissociate (pH 2.0) and re-associate (pH 8.0) the 24-mer assembly. However, to investigate if the PAS polymer may directly interact with the Dox molecules, we decided to remove the PAS polymer after Dox encapsulation and check it for Dox content. PAS polymer was removed using the specific matrix metalloproteases 2 and 9. After the cleavage and dialysis steps, HFt-Dox contained about 86 Dox molecules, indicating that a negligible fraction of Dox molecules could be bound by the PAS polymer. The samples produced were kept in sterile PBS at pH 7.4 and used for the following studies.

### 2.2. In Vitro Antiproliferative Effects of HFt-MP-PAS40-Dox Nanocarriers

**Figure 1:**
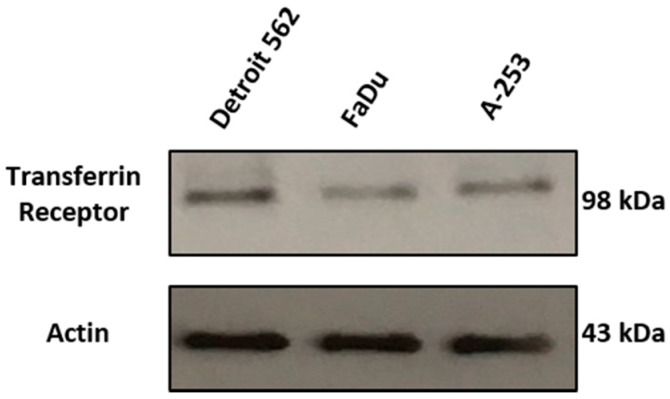
Transferrin receptor (TfR1) expression in the human HNSCC cell lines tested. β-actin was used as a loading control.

Efficacy of HFt-MP-PAS40-Dox has been demonstrated previously in pancreatic cancer xenograft model [18]. Herein, we decided to extend these findings to other cancers representing clear unmet medical needs such as HNSCC. HNSCC is now the seventh most common cancer worldwide, with a median overall survival for patients with recurrent and/or metastatic tumor remains less than one year despite modern systemic chemotherapy and targeted agents. The HFt system has the ability to be internalized by many types of tumor cells via the transferrin receptor 1 (TfR1). A wide number of tumor types (e.g., liver, lung, pancreatic, colon, cervical, ovarian, prostate and bladder, glioblastoma, breast, sarcoma, leukemia, etc.) express high levels of TfR1. First of all, we performed western-blot experiments to also verify the presence of this receptor on HNSCC cells of different origin: hypopharynx (FaDu), pharynx (Detroit 562), and submaxillary salivary gland (A-253) (Figure 1). All the HNSCC cells tested express TfR1 (Figure 1). We then assessed the killing ability of Dox-loaded HFt-MP-PAS40 on these cell lines.

**Figure 2:**
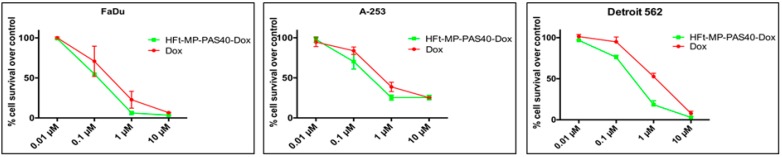
In vitro effects of HFt-MP-PAS40-Dox. Killing efficacy of Dox and HFt-MP-PAS40-Dox against human HNSCC cell lines. Cells were incubated for 72 h with different concentration of drugs. Cell viability was analyzed by CellTiter-Blue^®^ Cell Viability Assay. Mean ± SD (*n* = 3), experiments were performed in triplicates.

As shown in Figure 2, HFt-MP-PAS40-Dox was able to kill all the HNSCC cells tested in a dose-dependent manner even better than the naked Dox. This is remarkable, since in cell culture systems naked drugs freely diffuse into cells, whereas HFt-nanocarriers deliver the drug Dox by rate-limiting receptor-mediated uptake. This result can be ascribed to the considerable nuclear accumulation of the delivered drug, as revealed previously by confocal microscopy experiments [7,18,22]. The mechanism proposed by different groups included ourselves suggest that following TfR1 binding at cell surface, HFt-Dox carrier is internalized in an endosomal compartment where a mildly acidic (pH 5.5) favors some Dox release/translocation in the cytoplasm, diffusion to the nucleus, and initial DNA damage. In this way, Dox would then trigger a self-sustained, progressive nuclear translocation loop leading to extensive cell damage. Once in the cell nucleus, HFt is degraded releasing most of the Dox loaded. Likewise, our HFt-based canocarriers are likely to act like “Trojan horses” delivering high amounts of the drug right to the site of action of the drug itself.

### 2.3. Therapeutic Evaluation of HFt-MP-PAS40-Dox Efficacy In Vivo

**Figure 3:**
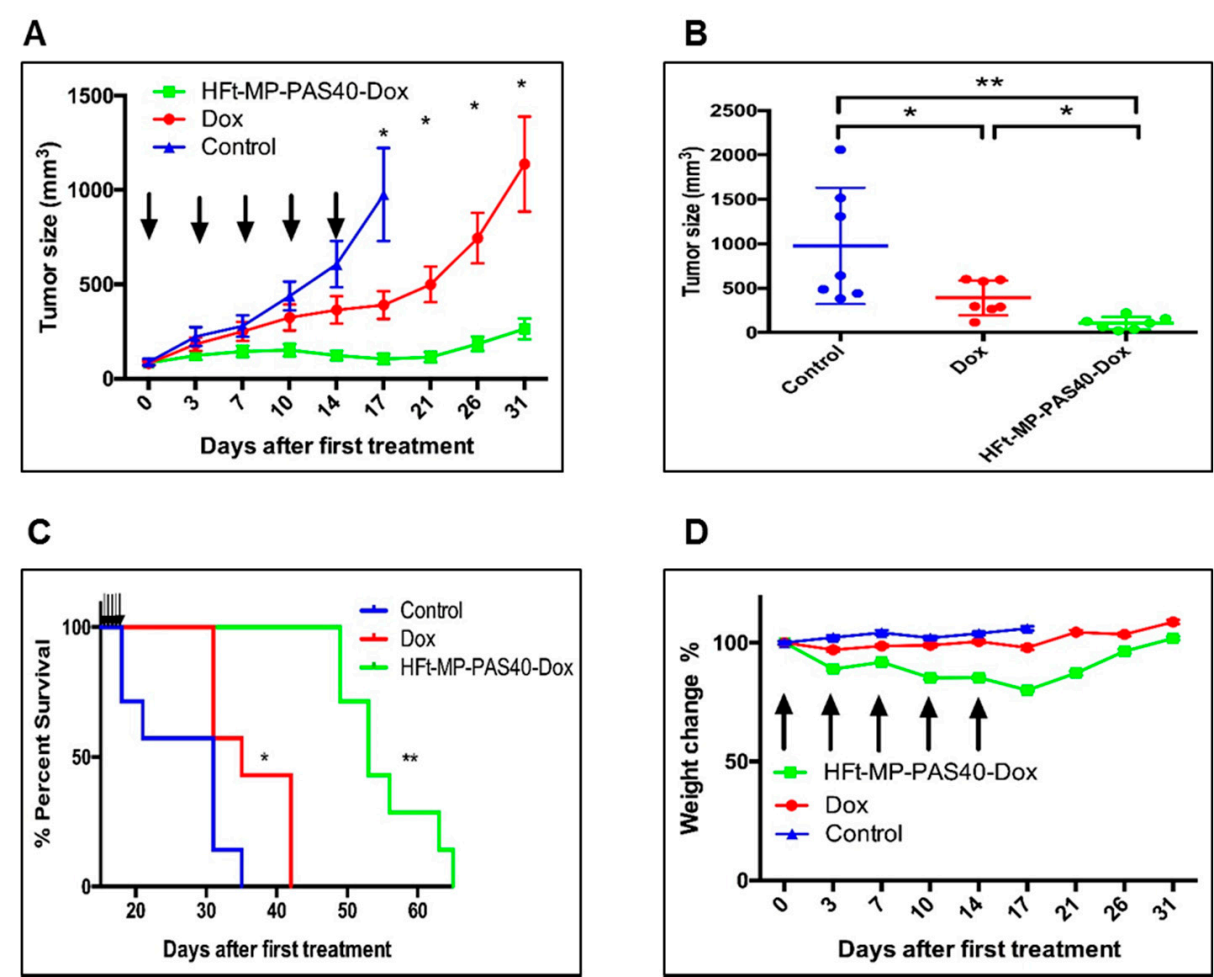
Anti-tumor activity of HFt-MP-PAS40-Dox in mice bearing FaDu tumors. (**A**) Tumor-growth curves for mouse groups are indicated. Student’s *t*-test is used to determine the statistical significance. * *p* < 0.05 and ** *p* < 0.001; (**B**) Single animal tumor sizes at day 17; (**C**) Survival curves of different animal groups bearing FaDu tumors. Animals were sacrificed when the tumor had reached a volume of ≥1500 mm^3^. Statistical analysis was performed by Log-rank test. Control vs. Dox * *p* < 0.05. Control vs. HFt-MP-PAS40-Dox ** *p* < 0.001. Dox vs. HFt-MP-PAS40-Dox ** *p* < 0.001. Arrows indicate the five treatments (5 mg/kg Dox equivalents); (**D**) Body weight changes of tumor-bearing mice after treatments.

The therapeutic efficacy of HFt-MP-PAS40-Dox was then evaluated in vivo in a model FaDu-derived xenografts. Three million cells were inoculated subcutaneously in nude mice and when tumors reached an average volume of 100 mm^3^ animals where divided in three arms (*n* = 7) and treated with a total of five injections (twice weekly). Mock treated mice were i.v. injected with 200 μL of PBS; for treated mice, the injected doses were normalized to the drug (i.e., 5 mg/kg for both Dox or HFt-MP-PAS40-Dox). The dose used in this in vivo setting is slightly below the reported maximum tolerated dose for Dox (MTD: about 6–7 mg/kg) [23]. As shown in Figure 3A, while free Dox treatment slightly reduced tumor growth, HFt-MP-PAS40-Dox treatment resulted in significant and lasting tumor growth inhibition. Of note, at day 17 from the start of drug administration, two out of seven mice showed nearly a complete remission (Figure 3B), resulting in a prolonged survival of 65+ days after the start of the treatment (Figure 3C). Overall, the median survival time for HFt-MP-PAS40-Dox-treated mice was 55 days versus 35 days for Dox and 30 days for saline control animals.

No differences in terms of body weight loss was observed in any of the treated groups (Figure 3D). This result resembles that reported by our group using a nude mice bearing xenogeneic pancreatic (PaCa-44) tumor model [18].

### 2.4. Tolerability of HFt-MP-PAS40-Dox

To assess the Dox-associated toxicity of HFt-MP-PAS40-Dox, we performed histopathological examination of liver and heart tissues derived from the efficacy studies. It has been reported that the liver is the main organ for ferritin accumulation, while heart is a key tissue due to the well reported cardiotoxicity associated to the Dox chemotherapy in clinical trials [3]. The animals of all treated groups show no evidence of liver injuries, as evaluated by IHC (data not shown).

**Figure 4:**
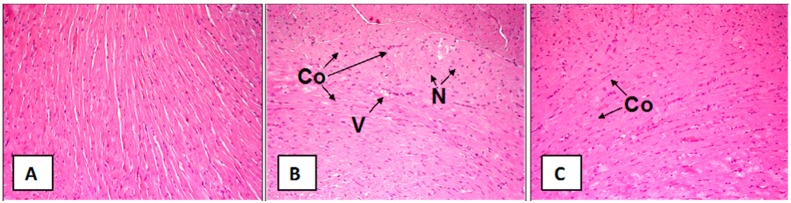
Representative images of the histologic evaluation of heart sections of animals from the control (**A**), Dox (**B**), and HFt-MP-PAS40-Dox (**C**) groups. A normal heart morphology is observed in (**A**). Diffuse blood congestion (Co), cytoplasmic myocyte vacuolization (V), and size variation in myocyte nuclei (N) are present in (**B**), whereas (**C**) shows only a light blood congestion (Co).

In the examined heart tissues, diffuse blood congestion, cytoplasmic myocyte vacuolization, and size variation in myocyte nuclei were observed in the Dox-treated group (Figure 4B) as compared to the control group (Figure 4A). In contrast, only a light blood congestion phenomena were observed for the HFt-MP-PAS40-Dox-treated group (Figure 4C).

Therefore, treatment with HFt-MP-PAS40-Dox nanocarrier can represent a promising option for anthracycline therapeutic regimens in cardiosensitive subjects as it did not display apparent anthracycline-related cardiotoxicity. These results well agree with those reported by other groups using HFt-Dox as nanocarrier [3,24].

In addition to the histopathological experiments, a single-dose tolerability study was performed. Healthy BALB/c mice were i.v. administered with HFt-MP-PAS40-Dox or free Dox at different mg/kg Dox equivalents doses and monitored for body weight and signs of distress as well. The maximum tolerated doses (MTDs) for these single injections were determined to be 7 mg/kg for Dox and 25 mg/kg for HFt-MP-PAS40-Dox. At these doses, no abnormal clinical observations or appreciable body weight loss (less than 5%) were observed. All these results demonstrate that the encapsulation of Dox into HFt-based nanocarriers greatly improves the drug tolerability and suggest that higher doses than the 5 mg/kg regimen used here could be exploited for future experiments.

## 3. Materials and Methods 

### 3.1. Cloning, Overexpression, and Purification of HFt-MP-PAS40-Dox Construct

The recombinant protein HFt-MP-PAS40 was expressed in *Escherichia coli*, purified, and quantified as previously described [18].

### 3.2. Dox Encapsulation in HFt-Based Nanocarriers 

**Scheme 1:**
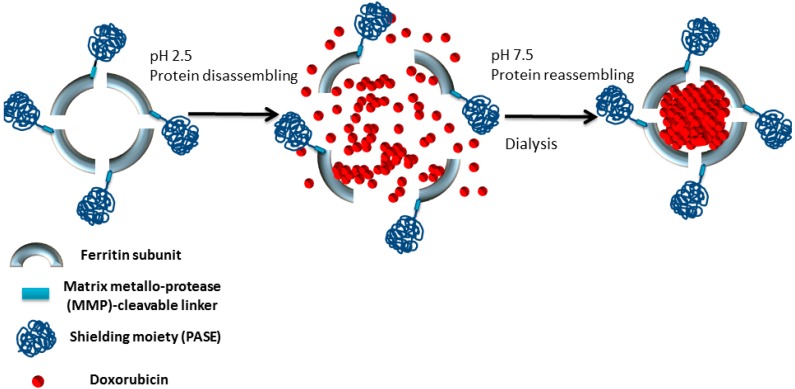
Schematic representation of the synthesis of HFt-MP-PAS40-Dox. For clarity purposes, only 4 out of the 24 modified HFt N-termini are shown.

The chemotherapeutic agent doxorubicin (DOX) (MedKoo Biosciences, Morrisville, NC, USA) was encapsulated using the previously reported HFt disassembly/reassembly procedure (Scheme 1) [4]. Briefly, solutions of HFt-MP-PAS40 in 0.1 M NaCl were incubated for 10 min at pH 2.5 (pH adjusted with HCl). Then, Dox was added to the solution at 300:1 molar ratio with respect to the protein. The pH was initially maintained at 2.5 for 5 min and then increased to 7.5 using NaOH. The resulting solution was stirred at room temperature for 30 min, filtered and dialyzed o.n. vs. phosphapte buffer saline (PBS) at pH 7.4 to remove unbound Dox. After the dialysis, centrifugation and concentration steps, protein and Dox content were then determined using the DOX molar extinction coefficient ε = 12,250 M^−1^·cm^−1^ at 485 nm. Dox content was also evaluated after PAS polymer removal. Enzymatic cleavage was performed as described previously using collagenase IV (Collagenase Type 4 containing MMP2 and 9, Clostridium histolyticum, Abnova, Germany) [18].

### 3.3. Antiproliferative Effects of HFt-MP-PAS40-Dox In Vitro 

FaDu, Detroit 562, and A-253 (ATCC, Rockville, MD, USA) head and neck cancer cell lines were seeded one day before treatment in 90 μL of complete medium in 96-well culture microplates. The next day, cells were incubated in triplicate with 10 μL of serial dilution of Dox and HFt-MP-PAS40-Dox. Afterwards, 72 h cell viability was investigated by CellTiter-Blue^®^ Cell Viability Assay (Promega Italia, Milan, Italy) according to the manufactory instructions.

### 3.4. Western Blot Analysis 

Whole cell lysates were prepared using ice-cold lysis buffer supplemented with protease inhibitors. Protein content was determined by Bradford method. Twenty micrograms of total proteins were loaded on a 10% SDS-PAGE and transferred to a nitrocellulose membrane. The membrane was blocked with 5% not-fat dry milk in PBS with 0.01% Tween 20 for 1 h at room temperature and then incubated overnight with primary antibodies: anti-trasferrin (TfR1) receptor used 1:1000 (Abcam, Cambridge, UK) and anti-β-actin used 1:40,000 (Sigma-Aldrich, Milan, Italy). Then the membrane was washed and incubated for 1 h at room temperature with the corresponding horseradish peroxidase-conjugated secondary antibody, diluted 1:20,000 (BioRad, Milan, Italy). Bound antibodies were detected using the enhanced chemiluminescent (ECL) method (PerkinElmer Italia, Milan, Italy).

### 3.5. Therapeutic Evaluation of HFt-MP-PAS40-Dox In Vivo 

Six-week-old female CD1 nude mice (Charles River Laboratories; Calco, LC, Italy) were injected subcutaneously in the right flank with 3 × 10^6^ FaDu cells resuspended in 200 μL of PBS. When tumors reached a volume of about 100 mm^3^, mice were randomized in groups of seven animals and injected i.v. with 200 μL of PBS, Dox or HFt-MP-PAS40-Dox. The treatment dose normalized to the Dox concentration was 5 mg/kg. Mice were treated five times twice a week; tumor volume was measured with a caliper and mouse weight was monitored. A tumor volume of 1500 mm^3^ was chosen as endpoint after which mice were sacrificed. Survival curves were derived from Kaplan–Meier estimates and compared by log-rank test (GraphPad Prism 5). Animal studies were performed according to a protocol approved by the Institutional Animal Care and Use Committee of University of Verona and authorized by the Italian Ministry of Health (Protocol No. 128/2014-B, 28/03/2014), and in accordance with the principles laid down in the European Community Council Directives.

### 3.6. Histology

When the tumor volumes exceeded 1500 mm^3^, animals were sacrificed. Mice heart and liver tissues were isolated and placed into 4% formaldehyde solution (Bio-Optica, Milan, Italy) for fixation, then dehydrated and finally embedded in paraffin. Sections were cut for hematoxylin and eosin (HE) staining and organ toxicity analysis.

## 4. Conclusions

To our knowledge, this report is the first to evaluate the antitumor effects of the novel stimuli-sensitive nano-ferritin containing doxorubicin (HFt-MP-PAS40-Dox) in any head and neck cancer malignancy. The pharmacological activity of the drug against a panel of different HNSCC cells was demonstrated by performing viability assays in vitro. The in vitro results showed that the anti–HNSCC effects of HFt-MP-PAS40-Dox were comparable or even superior to the naked Dox.

Strikingly, HFt-MP-PAS40-Dox displayed excellent therapeutic efficacy in the head and neck cancer model FaDu in vivo, reducing tumor growth and increasing animal overall survival significantly. The higher in vivo efficacy of our nanosystem could be ascribable to the combined contribution of active tumor targeting that exploits the selective TfR1 expression on HNSCC cells, and the increased delivery into the cell nucleus as observed previously.

From a drug tolerability point of view, acute toxicity and histopathological studies indicate that Dox is better tolerated when encapsulated inside the protein cavity. The maximum tolerated dose of HFt-MP-PAS40-Dox was about 3.5 higher than the free drug and the evaluation of heart tissues provided evidence for a safer profile of our nanosystem in terms of cardiotoxicity.

All the above findings provide support to carrying out additional preclinical studies on HFt-MP-PAS40-Dox either as single agent, possibly at higher doses, or in combination with other drugs in cancer therapy.
